# Intelligent Sensing to Inform and Learn (InSTIL): A Scalable and Governance-Aware Platform for Universal, Smartphone-Based Digital Phenotyping for Research and Clinical Applications

**DOI:** 10.2196/16399

**Published:** 2019-11-06

**Authors:** Scott Barnett, Kit Huckvale, Helen Christensen, Svetha Venkatesh, Kon Mouzakis, Rajesh Vasa

**Affiliations:** 1 Applied Artificial Intelligence Institute (A2I2) Deakin University Geelong Australia; 2 Black Dog Institute UNSW Sydney Randwick Australia; 3 Mindgardens Neuroscience Network Sydney Australia

**Keywords:** eHealth, e-Mental health, mHealth, digital phenotyping, personal sensing, smartphone, iPhone, software development, software framework, technology platform

## Abstract

In this viewpoint we describe the architecture of, and design rationale for, a new software platform designed to support the conduct of digital phenotyping research studies. These studies seek to collect passive and active sensor signals from participants' smartphones for the purposes of modelling and predicting health outcomes, with a specific focus on mental health. We also highlight features of the current research landscape that recommend the coordinated development of such platforms, including the significant technical and resource costs of development, and we identify specific considerations relevant to the design of platforms for digital phenotyping. In addition, we describe trade-offs relating to data quality and completeness versus the experience for patients and public users who consent to their devices being used to collect data. We summarize distinctive features of the resulting platform, InSTIL (Intelligent Sensing to Inform and Learn), which includes universal (ie, cross-platform) support for both iOS and Android devices and privacy-preserving mechanisms which, by default, collect only anonymized participant data. We conclude with a discussion of recommendations for future work arising from learning during the development of the platform. The development of the InSTIL platform is a key step towards our research vision of a population-scale, international, digital phenotyping bank. With suitable adoption, the platform will aggregate signals from large numbers of participants and large numbers of research studies to support modelling and machine learning analyses focused on the prediction of mental illness onset and disease trajectories.

## Introduction

There has been a recent explosion of interest in consumer digital tools across health care, such as smartphones. Mental health has been no exception [1]. Clinically valuable applications have been identified in depression and anxiety, suicidality, drug and alcohol disorders, ageing and dementia, and neurological disease. Through digital phenotyping [2,3] (or personal sensing [4]), behavioral signals, sensor data, and self-reported information gathered through smartphones, wearable sensors and smart home devices can be combined to elucidate the nature and clinical status of health conditions, such as depression [5,6], anxiety [7], and bipolar disorder [8-13]. These signals also promise better insight into the earliest signs of mental disorders, such as changes in sleep, social behavior, and cognitive function, and raise the prospect of robust, individual-level risk stratification and prediction [14]. Modern smartphones contain multiple sensors that may be used for both passive (ie, continuously running in the background without intervention) and active (ie, with direct input from the user) data collections. Individual sensors may be used for both, such as applying a device accelerometer to collect measurements from a stereotyped movement task while also recording circadian movement patterns.

Importantly, these digital tools can be designed to use gathered data to deliver and enhance interventions [2]. Techniques such as artificial intelligence can transform data into self-management recommendations concerning relapse or treatment adjustment, and can also drive interactive experiences, such as chatbot-based conversations [15,16]. The use of online and smartphone apps for the prevention and management of mood disorders [17], suicide [18], bipolar disorder [19], and the promotion of mental wellbeing has either already been established or is under active investigation and can deliver clinical outcomes comparable to face-to-face therapy [20-24]. Automation has created opportunities to reach global users in a timely way; creating new mechanisms to support health care users that do not rely on face-to-face services [25]. Data-driven tailoring also has the potential to address key challenges of poor engagement and meaningful use that are commonly seen in today’s apps [26,27].

Today, efforts to realize a vision of smart sensing and adaptive intervention design are fragmented and often narrowly focused. Despite enthusiastic efforts to build sensing and intervention apps, many appear to have limited potential for clinical translation because they are not backed by evidence of efficacy [28], or do not clearly satisfy clinical quality [29] and safety expectations (such as those concerning diagnostic accuracy [30]). Our own review suggests that the choice of data collected appears more often determined by technical ease rather than what is most important to understand physiological, neurological, and psychological processes [31]. There are also numerous questions of data privacy [32], acceptability [33], and the ethics of data collection [34] which have been fueled by high-profile public data scandals. Left unaddressed, these questions risk undermining public and professional confidence in this transformative technology area. Crucially, without large-scale public participation, much of the vision for digital phenotyping and adaptive interventions cannot be realized. It is becoming increasingly clear that a complete and comprehensive data platform is required to capture the breadth of available sensor data in a meaningful way. This itself is a challenge, given the need to specify the various functional modules required to support the range of available sensors without negative impacts on user experience and to meaningfully curate the data that is produced.

As a field, and at the beginning of what may prove to be one of the most useful ways we can help understand the nature, trajectory, treatment, and mechanisms of mental health disorders, we need to change the way we operate. Over the next few years, if appropriately and ethically acquired, we believe that the time has come to create an international digital phenotype bank. Its purpose will be to explore the relationship between behavior and the development, and subsequent trajectory, of common mental illnesses. It will also create the opportunity to develop personalized digital interventions that assist individuals in identifying and preempting periods of ill health. Because predictive analytics and related methods can efficiently leverage existing data [35], data consolidated in a digital phenotype bank promises a multiplier effect from reuse by clinical, research and, with appropriate governance, industry users.

However, to enable these research and clinical uses, signals data from potentially large numbers of participants must be collected, marshalled, and persisted. A key enabler of this vision is, therefore, the availability of scalable software platforms backed by appropriate technical architecture. This paper describes the design of a new platform for digital phenotyping intended to satisfy this requirement. We outline the design goals of the platform and highlight aspects that researchers may wish to consider when developing similar platforms.

## A Case for Shared Platforms

### Overview

The technology landscape surrounding the development of both digital phenotype data collection and data-driven intervention studies can be characterized by significant heterogeneity (of technologies, platforms, clinical problems, and research approaches [31]). Nevertheless, several common features stand out. These motivate our interest in the potential for shared data platforms and are summarized below.

### Most Digital Data Collection and Intervention Tools Are Custom-Built

Researchers in electronic mental health (e-mental health) often build custom digital solutions to support their studies. This typically involves creating study-specific mobile (or web) apps, often from scratch. These digital health research apps contain code for gathering study specific data from sensors, surveys, and custom workflows (such as games). Additionally, these apps must handle aspects such as user management, data transport to a secure server, data privacy, and ensuring that the technical solution adheres to the approved ethics protocol. This careful process of technical engineering to satisfy research and governance requirements must be repeated for every study.

### There Is a Significant Opportunity Cost for Creating Digital Solutions

Although individual research groups may achieve economies of scale through strategic development of shared code and the reuse of assets, the ability of other research groups to exploit these benefits is limited by the commitment of the originating researchers to make these assets available (eg, as open source repositories), to maintain them, and to provide support for their use. Researchers (especially those working outside electronic health [eHealth]) who may be interested in acquiring digital sensor signals may be essentially locked out because they have no access to enabling technology.

Efforts by mobile platform vendors to create reusable research tools (eg, Apple’s ResearchKit [Apple Inc, Cupertino, California, USA]) do not address passive sensing scenarios and do not solve the problem of researchers wanting to build tools that will run on both iOS and Android platforms, since vendor frameworks are not cross-platform compatible.

### Life Cycle Concerns Are Rarely Addressed

To guarantee participant experience, digital solutions must be supplied, set up, managed, and supported over the duration of the study. Significant effort and resources may be needed to address breaking changes introduced by updates to mobile operating systems and to debug issues experienced on specific device types (particularly for Android, where the device market is highly fragmented and where modifications to the device operating system by individual device manufacturers can create unexpected issues). The challenge of resourcing life cycle management is a potential contributor to the small numbers of research-backed health apps currently available in public app stores [28,36].

### No Common Data Standards Exist for Digital Phenotyping

A data standard is a consensus set of rules for describing and recording information to facilitate its analysis, reuse, and exchange [37]. A topical challenge for digital phenotyping is the extent to which contextual information may be needed to adequately interpret signals data. For example, device type, version, and power state, sensor characteristics (such as measurement precision), and user characteristics (such as height and age) may all affect the interpretation of sensing data. Data standards offer a means to ensure that this relevant information is consistently collected in formats that are useful for both initial and secondary analyses. A lack of common standards acts as a potential barrier to combining data from multiple studies, with time and effort needed for data wrangling (ie, the process of converting data into compatible forms). Shared platforms stand to help address the standards gap in two ways. Firstly, simply by coordinating the activity of different researchers, they operate as *de facto* standards providers since all data collected is governed by the same technical collection mechanisms. Secondly, as the digital phenotyping landscape matures, they are well-placed to implement any standards that are collaboratively developed by the research community. Other areas where standardization may be beneficial include the specification of data preprocessing steps and the methods used to derive summary metrics and features intended for machine learning.

### Good Data Governance is Challenging

Poor quality and privacy controls, including a lack of industry-standard safeguards such as appropriate encryption and access control, have repeatedly been identified in health apps available to the public [32,38]. Shared platforms may be better able to command the resources and expertise needed to monitor and respond to evolving governance risks than individual projects.

## Design Considerations for Digital Phenotyping Platforms

In addition to factors motivating interest in coordinated approaches towards digital phenotyping, there are several specific design considerations. In a recent discussion [31], we identified three priorities. We argued firstly for the need for universal (ie, cross-platform) technology that is accessible regardless of device type, to ensure equity of opportunity for both research participants in the present and the potential future users of clinical services that are built around digital phenotyping. Despite almost equal market share between the two major mobile operating system vendors (Android and Apple) in multiple economies [39], digital phenotyping platforms have historically focused exclusively on Android devices because of the relative ease of implementing passive sensing on these compared to Apple, whose app model does not allow continuously running background services (except in specific circumstances not directly relevant to digital phenotyping).

Secondly, we advocated for platforms that could not only efficiently support smaller pilot and exploratory projects (of the kind that have largely characterized digital phenotyping research to date [31]) but also larger studies running with potentially thousands of concurrent participants. We justified this requirement on the basis that, if a primary intent of digital phenotyping is *de novo* biomarker discovery for clinical grade uses, then there will need to be a step change in the discriminative performance of models being derived from digital phenotyping signals, which will likely require larger datasets [40]. Reported sensitivities and specificities in digital phenotyping classification studies to date rarely exceed 90%, despite the use of state-of-the-art machine learning methods. We have highlighted how improvements in test statistics will be needed to obtain clinically acceptable rates of false positives and negatives [31].

Thirdly, and relatedly, we identified a need for platforms that could support the aggregation and efficient transformation of collected data to: (1) support its secondary reuse, such as the creation of artificial intelligence–based methods for predicting mental health outcomes; and (2) enable future integrations with digital health interventions. For example, models derived from digital phenotyping data could be used to tailor the selection and timing of components delivered by adaptive interventions. Platforms supporting this kind of operational use case have potentially more stringent requirements around uptime and resilience than those used simply as repositories for research data.

We identify here two further design considerations. The first is that the use of smartphones as the primary data collection mechanism introduces multiple constraints which necessitate trade-offs during platform design. That is, there are competing characteristics, such as user privacy concerns, energy efficiency for extending battery life, hardware difference due to device fragmentation, and strict app development guidelines [41-43]. Because each of these constraints has relevance for research-relevant goals, such as being able to collect as much data as possible from a given user, there is a need for an explicit decision-making process during platform design.

Table 1 highlights the relationship between typical constraints and research goals, the tradeoffs that result, and potential design solutions. For example, there is an implicit trade-off between maximizing data collection volumes and potential negative impacts on user experience resulting from sensor-related battery drain and the use of data services to transmit collected data. For platform creators, design challenges include not only finding technical strategies that can optimize the efficiency of collection but also whether and how to include hard constraints that mitigate poor user experience (such as placing an upper limit on data collection frequency). Whether or not such strategies are necessary may depend, in turn, on the kind of operational model backing the platform. For example, where a platform is offered as a service to multiple research groups, the use of such constraints may be justified on the basis that negative user feedback arising from one study has the potential to affect the recruitment and participation of users in other studies, even if unrelated.

**Table 1:** Summary of the trade-offs between requirements, constraints, and resolution.

Research goal	Smartphone constraints	Trade-off	Potential design solution(s)
Readily accessible participant data	Protect user’s privacy by restricting access to data	Data availability versus user privacy	Strict adherence to mobile platform app guidelines captured in a reusable app development kit for app developers
Unified way to collect passive and active data	Smartphone vendor fragmentation creating platform-specific data collection challenges, such as continuous background sensing on Apple devices	High quality data versus Platform and device limitations	Tested and verified implementation for accessing sensor data that wraps platform-specific data collection strategies in a common interface
High resolution data collection strategy (eg, continuous high frequency sampling over many days)	Sensor usage limited to extend battery life Impacts on network responsiveness and user data costs if cellular networks used to upload large data payloads	High resolution data versus poor user experience High resolution data versus additional costs to participant	Custom communication protocol between mobile apps and core platform allows tailoring of frequency and duration of sensor sampling to manage energy and bandwidth impacts for users Platform enforces upper limits on data collection resolution Platform merges and schedules multiple requests to minimize impacts on users’ devices Support for customized scheduling of data uploads (eg, to use only Wi-Fi connectivity)

The second concerns the extent to which any platform enforces particular workflows on research users and data collection participants through its design. Even with modern software development approaches, assumptions about how users will perform key tasks are often incorporated at an early stage. As a result, there are limits to which the initial design can be modified [44], even with subsequent refactoring [45]. For example, if strong governance of collected data is a desirable outcome then it may be justifiable to introduce common platform mechanisms, such as requiring Institutional Review Board (IRB) approvals before data collection can be activated. However, our own experience as researchers also recommends against creating strong constraints on exactly what data research teams try to collect or the methods used for collection. One potential strategy for resolving this tension is to give study teams freedom regarding the design and function of client-side infrastructure (eg, data collection apps) while still mandating appropriate controls, including governance requirements, over server-side infrastructure where data are collected and processed for subsequent analysis. This approach has been successfully used in widely adopted software frameworks, such as those used to provide usage analytics on mobile apps.

## The InSTIL (Intelligent Sensing to Inform and Learn) Digital Phenotyping Platform

### Background

The InSTIL (Intelligent Sensing to Inform and Learn) platform is a new, cloud-based system for collecting active and passive sensor signals from both iPhone and Android smartphones.

### Platform Design Aims

The platform was designed with the broad research aim of improving understanding of the causes and trajectory of youth-onset mood disorders using digital phenotyping. Requirements analysis was informed by the issues discussed above and by considering, through discussion with representative stakeholders, what would be needed to support multiple multidisciplinary teams working in parallel to explore different facets of this research challenge. These imagined teams consisted of: (1) mental health and clinical researchers wanting to explore relationships between specific, digital phenotyping–derived signals and traditional mental health outcomes, such as GPS data and self-rated depression scores, within observational studies of their own design; (2) researchers wanting to combine (with consent) collected datasets for secondary analyses, linkage, and machine learning; and (3) intervention designers wanting to consume digital phenotyping data in some way to tailor or optimize new mental health interventions. It was assumed that teams would not necessarily be from the same institution and, although aligned with the overall research aim, would not necessarily focus on the same study populations.

From this analysis, we identified three interrelated design objectives. Firstly, the platform should support high rates of reliable data ingestion (ie, the collection of high-resolution data from thousands of users without loss). This objective reflected both the identified need for, and research interest in, larger-scale digital phenotyping studies, as well as specific consideration of the data volumes required to establish a digital phenotyping bank. Platform design was influenced by this more than any other objective, reflecting the impact that performance and robustness requirements have on software architecture [46]. Secondly, the platform should be flexible enough to support the requirements of multiple studies, including those not based in mental health. In other words, the system needed to be flexible enough that researchers could adapt the platform to the specific requirements of their study while still benefiting from software reuse. Thirdly, the platform design should seek to minimize operational costs. Research teams often operate with constrained budgets with minimal allowance for the developer and operations teams that would be typical of such platforms in a commercial setting. This objective informed the ultimate decision to design certain platform components as shared backend services, thus removing the need for individual researchers to carry the risks and costs of initial setup and creating a potential mechanism for formal operational support in the future. Success in meeting this third objective is being assessed through cost modelling incorporated into a randomized controlled trial [47] that is using the platform for data collection. This will be reported in a future analysis.

### Common Research Workflow

**Figure 1:**
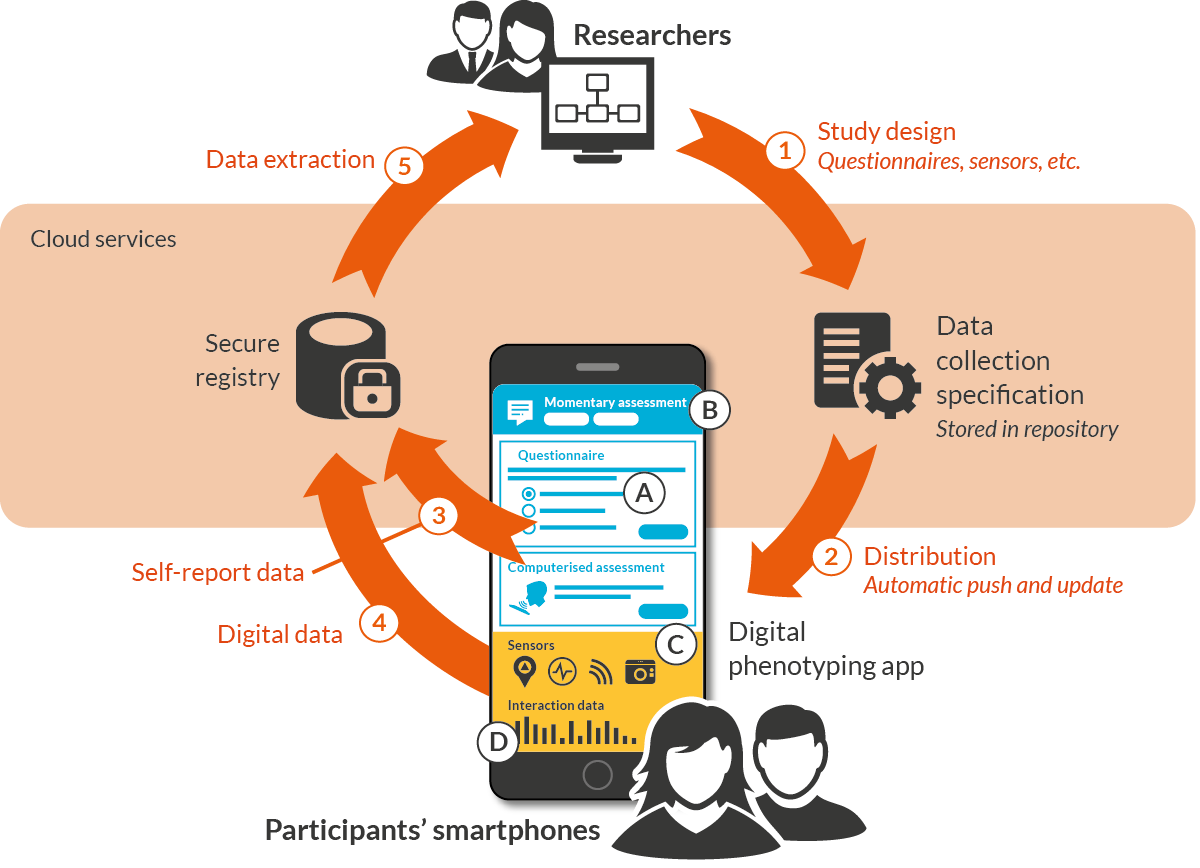
InSTIL Common research workflow. The figure shows how the platform supports a sequence of key activities for researchers involved in digital phenotyping. InSTIL: Intelligent Sensing to Inform and Learn.

From the requirements gathering process we identified a common research workflow (Figure 1), which is a stereotyped sequence of actions for the acquisition of digital phenotyping data from study participants. This workflow incorporates the following steps:

1. Researchers specify the study design, define which questionnaires and sensors are required to deliver a trial or set of clinical measurements and, optionally, how these are integrated with any intervention components, such as self-guided therapy. This specification is then hosted in a secure online repository.
2. When the study commences, the specification is automatically distributed to users’ devices. The same mechanism can be used to update the specification throughout the study.
3. The platform app development kit generates a range of data streams, as required. This includes self-report data streams, which consist of:
  Self-report questionnaires and assessments. Self-report data can include single questions, standard instruments (such as the Patient Health Questionnaire [PHQ-9]), structured tests (such as response-time measurements) and recordings from sensors (such as voice samples); and
  Ecological momentary assessments. These include quick-fire questions generated in response to temporal or contextual cues (for example, when a user awakes), to generate ecologically valid data.
4. There are also digital data streams, which might include:
  - Passive sensing, which runs silently and continuously to collect high resolution data about location, activity, and social interaction from the user. A contemporary smartphone may typically contain: an accelerometer, gyroscope, compass, barometer, light sensor, GPS receiver, microphone, camera, as well as Wi-Fi and Bluetooth interfaces that can be used to detect proximity to other users and devices. These sensor types are all routinely available for digital data collection.
  - Device utilization data, which tracks ‘digital exhaust’, such as time spent in apps, phone calls and text messaging usage, as well as potential markers of cognitive function, such as typing speed. The app development kit automatically manages potential barriers to data collection, such as user battery life and limited connectivity, through smart scheduling and caching.
5. Researchers can start to extract registry data as soon as it is received, accelerating analysis, permitting study designs that involve expert feedback, and allowing any data collection issues to be identified and addressed early in the research process.

The workflow makes explicit several user requirements that informed platform design. Firstly, and reflecting the status of digital phenotyping as an emerging research area, an iterative “test, analyze, test” approach is likely to be the norm, with the timing and focus of digital phenotyping data collection revised through a sequence of sub-studies. This requirement drives the cyclical graph shown in Figure 1. Any iterative approach to data collection must nevertheless be balanced with good study governance requirements such as data integrity and versioning.

Secondly, researchers who are faced with practical study issues, such as unforeseen protocol adjustments or managing participant compliance, will expect the flexibility to amend data collection parameters on demand. This gives rise to the concept of a dynamic, study-specific, data collection specification that can be updated and disseminated to participant devices as required.

Thirdly, support for acquisition of nondigital phenotyping data types is essential to enable key uses cases, such as the need for self-report and outcomes measures to provide labels against which to train digital phenotyping models using supervised machine learning.

Finally, despite the need for study-specific software development work (eg, engineering apps to satisfy specific data collection, intervention, and user experience requirements), there is still value in common, researcher-facing software tools to support routine administrative tasks such as scheduling, monitoring participation, and data extraction. Relatedly, the ways in which researchers will use collected data, whether for statistical analysis, machine learning, or intervention tailoring, are diverse enough to limit the value of single mechanisms for visualizing and manipulating data. Instead the focus should be on providing ways to efficiently extract collected data in useful formats and slices (ie, subsets of users).

### Platform Components

**Figure 2:**
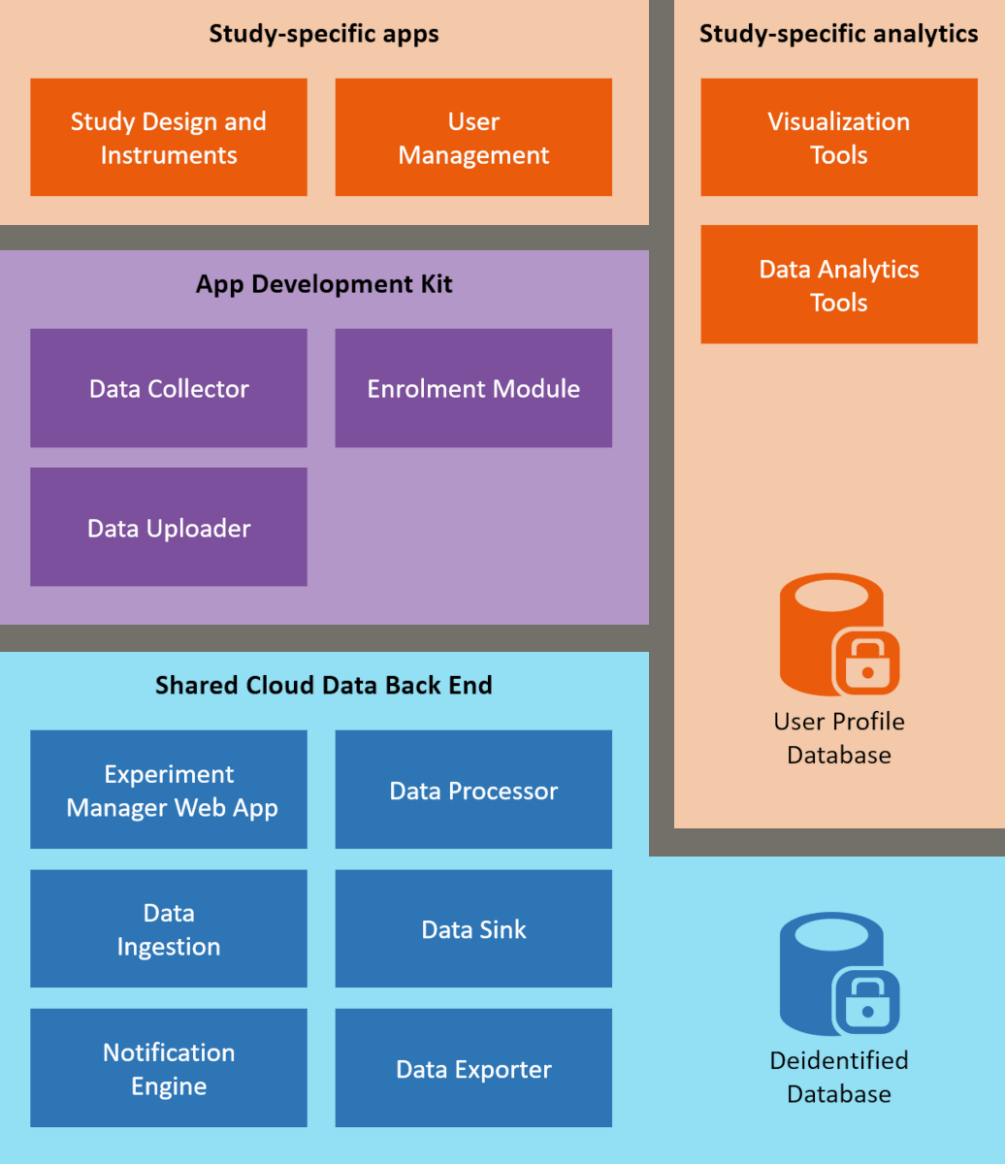
Platform architecture. Architecture diagram showing the digital phenotyping platform consisting of reusable components (purple and blue boxes) and components that need to be custom built for each study (orange).

The resulting platform consists of a set of reusable software components embedded within a common architecture (see Figure 2 and summary in Table 2). The architecture is consistent with identified requirements for big data platforms, specifically those of distributed computation and big data storage [48]. Together, these components: (1) enable the collection of passive and active sensor data (and other arbitrary data types); (2) enable secure storage of deidentified data; (3) provide dynamic control over the frequency and types of data being collected; (4) maintain the provenance of data by ensuring source information is recorded consistently; (5) provide a robust synchronization protocol to reduce the risk of data corruption during transfer; (6) record data in a standardized and stable data format to permit replication and auditing; and (7) offer a common data export and management method. The components shown in Table 2 have been designed to adhere to the software engineering principle of separation of concerns [49], where each component focuses on a well-defined functional process to minimize repetition of code, maximize reusability, and simplify maintenance or further development.

**Table 2:** Description of architecture components.

Component	Description
Study design and instruments	Questionnaire, data collection strategy, and the frequency required for a specific experiment (active data).
User management	Some studies may need to reidentify users or provide custom authentication to ensure that only specific participants join the study, and this component enables custom apps to provide that functionality.
Visualization tools	Integration endpoint for third party data visualization tools (eg, Power BI, Tableau, or Quicksight).
Data analytics tools	Integration endpoint for big data tools to provide analytics and support machine learning techniques.
User profile database	Database for storing study specific details. This store is independent of the platform, thus preserving the separation between identifiable data (held in this database) and anonymized data (held in cloud data stores).
Data collector	Component responsible for extracting passive data from the mobile device and storing it locally prior to uploading the data to the platform.
Data uploader	Component responsible for uploading data to the cloud backend. Fault tolerance and automatic resume-retry supported.
Enrolment module	Responsible for enrolling a participant and device with a specific experiment.
Experiment manager web app	Dedicated component responsible for creating a new experiment with all the details specific to the study.
Data ingestion	Accepts raw data from the mobile devices and triggers the data processor to store all data for the user.
Data processor	Backend component responsible for transforming the data to the Data Sink component.
Data sink	Component responsible for storing the study data in a database.
Notification engine	Triggers notifications to be sent to the mobile apps to ensure that they continue to collect the appropriate data required for the study.
Data exporter	Module that researchers can use to extract data from the database in a standardized format.
Deidentified database	Core database that stores all deidentified data collected from an app.

**Figure 3:**
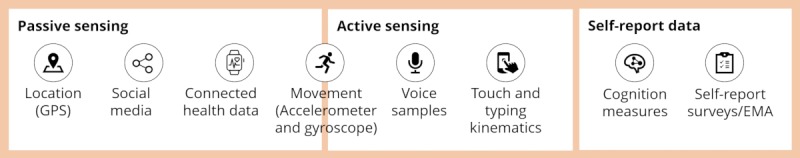
Supported data collection types. EMA: ecological momentary assessment.

Platform components are divided between a mobile app development kit and shared cloud-based data backend. The app development kit is available for both iOS and Android and is intended to expedite development of new digital phenotyping client apps. The app development kit reflects our skepticism that any fully-fledged data collection app can meet the design, content, and user experience requirements of any given study. Instead, native libraries included in the kit provide drop-in passive and active sensor data collection capabilities (summarized in Figure 3), and separate error-tolerant and secure upload management for collected data. These libraries allow any app to be augmented with digital phenotyping capabilities, while leaving researchers full control over the user-facing interface and interaction design. The app development kit also allows researchers to exert fine control over energy and data impacts associated with sensor utilization and data upload.

**Figure 4:**
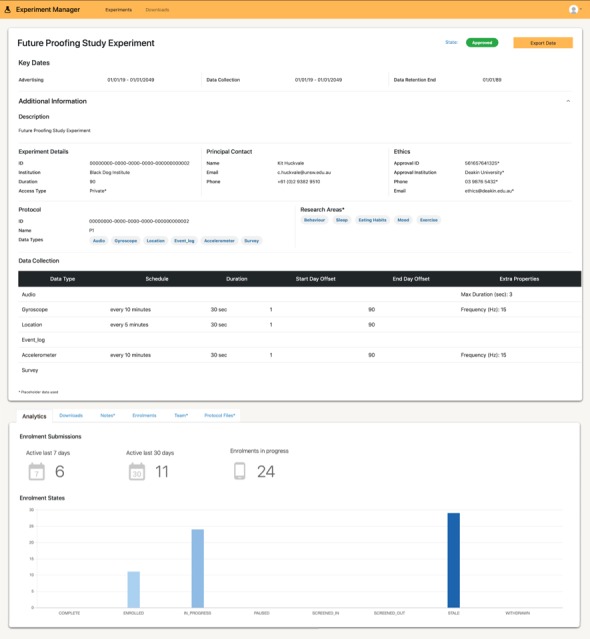
Experiment manager interface. Screenshot of the Experiment Manager application showing a summary of a study, including the data being collected (top half), and the enrollment status for the participants (bottom half).

The common data platform provides endpoints to authorize, ingest and securely store data uploaded by apps using the app development kit, allows researchers to dynamically configure the behavior of data collection apps, makes collected data available to researchers for further analysis, and maintains persistent audit logs of data upload and access for governance purposes. Although individual studies can set up and run their own instance of the common data platform, it is principally designed to support the use case where it can be hosted and offered as a service to multiple concurrent projects. The design is intended to reduce the learning curve, costs, and risks borne by users new to the platform who can instead focus on the client-side data collection experience. In our solution, researchers interact with the platform through Experiment Management, a web app (interface shown in Figure 4) which allows them to specify data collection configurations, monitor data collection progress, and download data collected from previous experiments. The platform supports the creation of custom study designs (questionnaires, interactions, etc) and flexible data collection protocols (nightly uploads, type and frequency of sensor data, etc). It also acts as a formal store for audit-relevant information about study permissions, such as documents received from an authorizing IRB. Without this information, the study cannot commence data collection.

### Platform Features

#### Support for Passive Sensing on Both iOS and Android Devices

The platform app development kit provides native libraries to support app development on the devices of both major platform vendors. The ability to schedule passive sensing on Apple devices differentiates ours from most other digital phenotyping platforms. The provision of a kit ensures that researchers do not need to reimplement best practices associated with privacy and security in a mobile cloud computing environment, such as appropriate authorization and encryption during data upload [50].

#### Strategies to Maximize Passive Sensing Completeness

A principal challenge for passive sensing on smartphones is to maximize the proportion of desired data points that are sampled as planned and successfully returned for analysis. Although data upload scheduling and error tolerance is an important modifier of data completeness, a major barrier concerns reliable sampling (ie, the initial acquisition of data from the desired sensor at the desired time).

Concerns around data privacy have led mobile platform vendors to introduce restrictions designed to give users greater control over how sensor data are collected and used. For example, mobile apps must now explicitly obtain (and in some cases periodically reobtain) user permission to collect GPS location data. These permission mechanisms are platform-specific and subject to change. To address these challenges, our development kit provides support for a flexible onboarding mechanism to guide users through the process of setting appropriate permissions, configuring whether cellular data can be used for data upload, and configuring hooks for notifying where permissions have been denied or revoked. Fault tolerance means that the kit handles such cases gracefully, continuing to collect other sensor types even if one sensor no longer has permission.

Another limitation arises from concerns about the potential impact of continuous background sensing on system performance and battery drain adversely affecting the end-user experience [43]. Platform vendors limit the amount of data that can be collected passively, even closing apps that are energy inefficient or have not been recently activated by the user. To work within the constraints imposed by the platform vendors, our communication protocol relies on periodically waking up the mobile apps using silent background notifications. This approach allows a common mechanism to be used on both Android and iOS but is reliant on network connectivity. The efficacy of these strategies in maximizing data completeness is the subject of current investigation and we intend to report on it in a future manuscript.

#### Extensible, Low-Cost Data Ingestion Pipeline

Data management has shown to be a core consideration in large-scale, mobile app ecosystems [51]. This principle influenced the decision to make the data ingestion pipeline hosted by the cloud platform essentially agnostic to the form and type of data uploaded from users’ devices. Instead, uploads are required to be decorated with metadata, which encodes information type and shared parameters, such as time of collection, in a standardized format. This flexibility means that support for new sensor types or data sources, or changes to the form in which data are collected, can easily be introduced to data collection clients without impacting the function of the cloud-based ingestion pipeline. For example, a research group wanting to extend the app development kit to collect high volume data from a wearable sensor could do so and continue to use existing data upload mechanisms without requiring changes to the cloud architecture. This flexibility is intended to expedite this kind of development and reduce the associated costs for both research users and platform operators. The use of standardized metadata means that analytics functions, such as data submission rates, can easily be derived from submitted data even if new types have been added.

In addition to extensibility, the data ingestion pipeline is designed to minimize overheads associated with data acquisition by performing minimal computation over the ingested data. For example, data validation and compression are not performed by the pipeline but are, instead, a client-side concern supported by the app development kit. By distributing computation to client devices, this reduces potential bottlenecks and consequent costs for the common platform.

#### Strongly Typed, Client-Side Data Collection

While the data ingestion pipeline is type-agnostic, the app development kit implements a core set of strongly typed schemas for capturing and persisting common sensor data and related data types. Schemas are currently available for GPS, accelerometry, gyroscope, audio, longitudinal event log, and questionnaire data and can be freely extended by users of the kit. This combination of an agnostic pipeline with client-side conventions for storing data represents a design trade-off between the flexibility to extend and adapt the platform and the ability to reuse, transform, and combine data (which recommends the use of common data standards). As a result, research users of the platform that implement the development kit can use the provided data types and be reassured that the data they collect will be compatible with existing datasets as well any visualization/analysis tooling developed for the platform, while those who want to adapt the data formats can do so freely.

#### Privacy-Preserving Architecture

Consumer privacy law and research ethical guidelines create strict requirements about data handling and management [52,53]. Considerations such as data privacy, which jurisdiction the data can be stored and transferred to, and system security are all relevant governance concerns addressed by the platform. For example, the selected cloud/provider architecture allows us to assure (with suitable configuration) that data belonging to each research study are appropriately segregated and will be stored only in a single legal jurisdiction, while the common data platform enforces a requirement for IRB approvals to be lodged prior to starting data collection.

**Figure 5:**
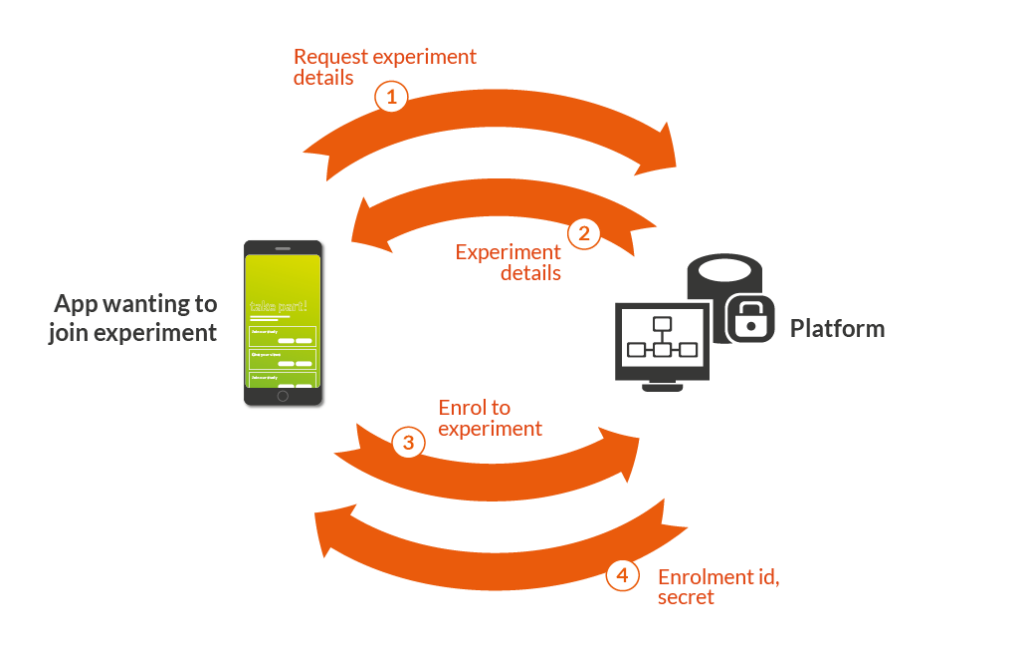
Privacy enforced enrolment protocol. Protocol for communication between the digital phenotyping app and the cloud backend supporting passwordless authentication.

A distinguishing feature of the platform is that all user data contributions are anonymous by default. This is achieved using a custom anonymous authentication mechanism (see Figure 5) which is provided as part of the app development kit and enforces contractual anonymity within the mobile app. We recognize, however, that for certain studies it may be necessary to identify and follow up with named individuals to augment or verify results or deliver other intervention components. The platform supports the case where participants do need to be identified, but this requires explicit additional steps to be taken by developers (in addition to appropriate ethical permissions) to cache the unique enrollment identifier generated for every participant. To do this, developers must create their own linkage key store. This is, by definition, separate from the data stores used by the platform, preserving anonymity in the unlikely event that the platform stores are compromised.

**Figure 6:**
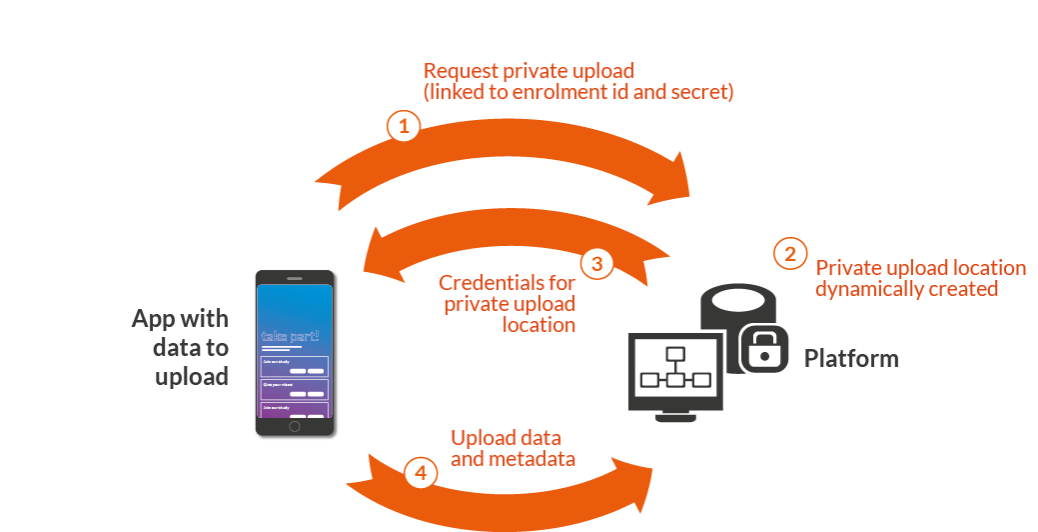
Secure data upload protocol. Protocol for communication between the digital phenotyping app and the cloud backend involving anonymous data collection and dynamically created upload locations.

A second feature is the use of public key-based mechanisms as a strategy to authorize and secure the time-limited transfer of user data payloads to the platform. This mechanism (summarized in Figure 6) guarantees that uploaded data are uniquely allocated to the user that initiated the upload, minimizing the potential for unauthorized access, upload spam, or mistakes in data allocation during analysis. Together, these strategies mean that there is no situation during routine platform operation in which identifiable information is stored in the same cloud environment as user response data.

### Technical Implementation Overview

A central tenet of the technical implementation was to leverage existing open source components wherever possible. Due to the scalable requirements of the platform, which is the eventual ability to support tens of thousands of concurrent users, the platform was built on top of the containerized platform Kubernetes (Google LLC, Mountain View, California, USA). Selecting a containerized solution simplified the processes of integrating existing tools into the platform. These included Elasticsearch (Elastic NV, Amsterdam, the Netherlands) for efficient information retrieval, Kafka (Apache Software Foundation, Forest Hill, Maryland, USA) for constructing data pipelines, Zookeeper (Apache Software Foundation, Forest Hill, Maryland, USA) for coordinating distributed components, Kibana (Elastic NV, Amsterdam, the Netherlands) for visualization and reporting, Redis (Redis Labs, Mountain View, California, USA) as a central data store and PostgreSQL (PostgreSQL Global Development Group, Berkeley, California, USA) for data persistence. Scripts are provided to configure, deploy, and run these components on Google’s Cloud Platform (Google LLC, Mountain View, California, USA) (although the platform will run on any cloud environment that supports Kubernetes). The applications that make up the cloud backend are written as Java microservices using the Spring Boot (Pivotal Software, San Francisco, California, USA) web framework. Digital phenotyping apps use platform specific native programming languages, such as Swift for Apple’s iOS and Java/Kotlin for Google’s Android.

### Future Development

A principal task for future development is to identify where shared value can be created for users once data have been received by the platform. While we expect that most researchers will expect direct access to raw data, there may be tasks, particularly those that are either computationally expensive or technically sophisticated, which it would make sense to be offer as platform services. For example, feature computation or data reduction techniques could be offered as options within a standardized processing pipeline, minimizing set-up burden for platform users while offering potential assurance about the standardization of the datasets that are produced. Work is underway to explore the scope, feasibility, and value of this kind of preprocessing.

Additional work is also required to enhance error handling logic for the platform. To achieve high data throughput, validation logic (ie, steps to verify the correctness of incoming data) within the ingestion pipeline was kept to a minimum. While this pipeline can theoretically support efficient validation, such as through asynchronous workers operating over accumulated data, the current architecture does not support mechanisms to propagate identified validation errors (eg, poor sample quality) back to mobile clients. This introduces potential latency into the process of identifying data quality issues and shifts the burden of problem-solving onto the research team as a manual process, which may be unfeasible in studies with large numbers of participants. Future updates will introduce mechanisms to trigger app development kit hooks when validation issues are discovered, providing a potential route for data collection clients to respond and fix any identified validation problems without manual intervention. For example, in a study collecting active voice samples, a collected sample that fails a quality check could trigger the platform-associated mobile app to ask a participant to provide a second sample.

We also recognize an ongoing requirement to review and strengthen approaches to safeguarding participant confidentiality. While the platform design ensures that traditional identifiers, such as names and contact details, are not collected, we acknowledge the risk that certain digital phenotyping data types, such as GPS, are intrinsically identifiable [54]. Potential platform-enforceable strategies to manage this risk include: holding data in temporal escrow to prevent individuals’ current location being identified, remapping GPS data to an alternate reference frame so that relationships between points, but not their absolute location, are preserved, and providing pipeline tools to preprocess data into features or labels on behalf of researchers to avoid the need for teams to handle raw data. Each of these involves potential trade-offs between risk management and analytical value [55]. For example, remapping GPS to an arbitrary coordinate system limits the potential for semantic labelling of known locations. We aim to explore the feasibility and acceptability of these kinds of strategies in relation to both GPS and other sensor data types in the next phase of work.

Finally, a process of technical refinement (currently underway) aims to simplify the existing architecture to: (1) eliminate unnecessary components which nevertheless confer operational costs; and (2) reduce the effort required to extend the platform for study-specific modifications. Currently, binary data such as audio, video, and image files are stored in Elasticsearch, enabling all data to be indexed for efficient searching. In later development phases, blob storage will be used to house binary data and only metadata describing the files will be indexed, with the aim of reducing data processing costs. This shift may also make it possible to replace Elasticsearch with a simpler data store, such as a relational database, potentially reducing the skills required of the team operating the platform.

## Discussion

In this paper we describe the design of a scalable and governance-aware platform for the acquisition of sensor data from consumer smartphones, for the purposes of digital phenotyping. The InSTIL platform provides a new suite of tools for the development of digital phenotyping research studies. The platform is currently being used to run studies at Black Dog Institute and Deakin University, and will ultimately be made available for research and public use. It sits alongside several established and emerging technology platforms for digital phenotyping that include Aware [56], Beiwe [57,58], EARS [59], Purple [60], Monsenso [61], Passive Data Kit [62], and RADAR-base [63]. InSTIL differs from these existing platforms in several ways: (1) it supports passive sensing on iOS, unlike EARS, Purple, and RADAR-base; (2) it runs sensing without using special permissions that may prevent apps from being deployed via public app stores, unlike Aware (albeit at the expense of certain sensor streams, such as Bluetooth proximity tracking); (3) and its hosted server model means that research teams do not have to worry about setting up, securing, and maintaining the server infrastructure needed to run digital phenotyping studies, unlike Passive Data Kit. Our platform appears to be conceptually like Beiwe as a service [64], adopting similar positions on issues such as participant anonymization [65]; however, InSTIL differs by pairing its hosting model with a software development kit, enabling custom apps to be built for each project rather than offering standard data collection apps (as Beiwe does). Each approach has pros and cons concerning the ability to tailor user experience versus development cost and timelines.

InSTIL is also an enabler for a research vision that seeks to establish an international digital phenotype bank to pool sensor signals data from a population-scale longitudinal cohort. This initiative creates the opportunity to link digital phenotyping data to ground truth data, including access to DNA information, hospital records, and educational outcomes, to sensor data within individual trials, projects, and experiments. So far, platform development has focused primarily on technical aspects of data collection, including the requirement to support the volume of incoming data that would be necessary for a population scale bank (potentially millions of data samples, per participant, per year). The data collection platform is not the bank, however, and we recognize that substantial additional work will be required to address technical, governance, and operational issues around efficient and secure data sharing (particularly across international boundaries), participant recruitment, and management.

From a technical perspective, there are several insights arising from the development process which may be relevant to other researchers involved in similar digital phenotyping or other platform-scale efforts. Firstly, for managing even high-volume data ingestion scenarios, a simple publish-subscribe messaging queue may suffice. In retrospect, our selection of Kafka as the queue management technology, while not creating any active roadblocks, now provides few benefits. While Kafka can perform persistent queries on the queue itself and replay data streams, these features add limited value in the final system. Second, the initial idea of simplifying the architecture by using a comprehensive search engine (Elasticsearch) for all different types of data proved to be unnecessary, as the principal data storage format ended up being JSON. A simple document storage database or a relational database system with JSON indexes would be enough. Even where binary data are being collected, such as audio samples, there is no specific use case for holding these in an indexable store. Rather, it is the outputs from analysis of these data packets (eg, machine learning/algorithm-derived labels and annotations) where indexing may be helpful, and since we propose that these outputs be in JSON format, these too can be kept in a relational or document store. Together these issues highlight the challenge of accurately forecasting appropriate technology selection in complex projects, particularly those developed using iterative approaches. Finally, building and maintaining a digital phenotyping platform requires a thorough understanding of the limitations and constraints put in place by the mobile platform vendors. This is a challenging task as mobile operating systems are frequently updated, often with breaking changes. For example, changes to the latest version of iOS significantly reduced the ease with which passive location data could be continuously collected.

In this paper, we also highlighted the trade-offs inherent in designing data collection infrastructure that runs on users’ own devices, which researchers have limited control over and where inconvenience must be minimized. In addition to these user-facing tradeoffs, the overall platform architecture itself represents a trade-off between the desire to give researchers maximum flexibility in configuring data collection to suit their research and user experience requirements, while minimizing the costs of completing technically challenging tasks such as passive sensing and a secure data upload. We use the client-server divide to orchestrate this trade-off, with researchers given freedom on the app (client) side while being supported by a common (server) backend with minimal scope for study-specific changes. Work is now needed to critically evaluate the value of this strategy, particularly since we have already seen evidence of how it might be challenged. While developing the app development kit, the team discovered that the iOS App Store review process prevents apps from collecting location data passively without surfacing this information in a feature that directly benefits the end user. A design question arises, then, as to whether the platform should provide native visualization of GPS location data in some user-facing format (which may require technical sophistication to achieve), or whether this should be a study-specific concern. As the platform is designed to be useful across many different studies, implementation of such a feature may be beneficial but comes with development and maintenance costs. It seems likely that, should the platform become more widely used, a prioritization mechanism will be needed to select features for inclusion in the development kit/backend. We also recognize that the putative benefits of a common and shared infrastructure are largely linked to the ability to provide operational support to projects. As a result, the next phase of platform development will also focus on the feasibility of different operational and governance models.

Although in the early stages of use, our vision for this new science of behavior [2] promises benefits for health care, digital health industries, and science. For health care, it provides the means for users to learn more about their mental health, to be able to, once sufficient work has been completed, anticipate when their health may be at risk, and be able to access techniques and interventions that have proved successful for themselves and for many others. Ultimately, this work is aimed at optimizing health care and alleviating disempowerment and emotional toil. For the fast-growing digital industry, it raises the possibility of new commercial opportunities, such as tools for health insurers that incentivize health behaviors through passive monitoring. For scientists, a collaborative platform to generate, combine, and use signals reduces the opportunity costs of digital phenotyping research and opens the door to new multidisciplinary collaborations.

There is some understandable skepticism about the potential of digital sensor signals to provide meaningful data to help manage mental illness. Our own uncertainties have been reduced, in part by the success of modern machine learning methods in tackling previously intractable classification problems in multiple domains, by promising results from early digital phenotyping studies [31], and by evidence supporting the existence of prodromal phases with behavioral correlates for common mental illnesses [66,67]. If these changes can manifest in sensor data streams, then they can be detected and modelled. Ultimately, we will not know if this can be a cost-effective, acceptable, and robust approach unless we try. A collaborative digital phenotyping platform, open to multiple users working in parallel, will be critical to answer this question quickly.

## Abbreviations

eHealth: electronic health
e-mental health: electronic mental health
InSTIL: Intelligent Sensing to Inform and Learn
IRB: Institutional Review Board
PHQ-9: Patient Health Questionnaire
